# Evaluation of Antimicrobial Activity of Triphala Constituents and Nanoformulation

**DOI:** 10.1155/2020/6976973

**Published:** 2020-08-02

**Authors:** Ziad Omran, Ammar Bader, Amalia Porta, Thierry Vandamme, Nicolas Anton, Zeyad Alehaideb, Hamdi El-Said, Hani Faidah, Abulrahman Essa, Antonio Vassallo, Majed Halwani

**Affiliations:** ^1^College of Pharmacy, Umm Al-Qura University, Makkah, Saudi Arabia; ^2^King Abdullah International Medical Research Center, King Saud Bin Abdulaziz University for Health Sciences, Riyadh, Saudi Arabia; ^3^Dipartimento di Farmacia, Università Degli Studi di Salerno, Fisciano, Italy; ^4^University of Strasbourg, Faculty of Pharmacy, Strasbourg, France; ^5^Department of Medical Genomics, King Abdullah International Medical Research Center, King Saud Bin Abdulaziz University for Health Sciences, Riyadh, Saudi Arabia; ^6^Department of Medical Microbiology, Faculty of Medicine, Umm Al-Qura University, Makkah, Saudi Arabia; ^7^Al-Noor Specialist Hospital, Makah, Saudi Arabia; ^8^Research Centre, King Fahd Armed Forces Hospitals, Jeddah, Saudi Arabia; ^9^Dipartimento di Scienze, Università Degli Studi Della Basilicata, Potenza, Italy

## Abstract

The prevalence of nosocomial infections due to multidrug resistant (MDR) bacterial strains is associated with high morbidity and mortality. Folk medicine and ethnopharmacological data can provide a broad range of plants with promising antimicrobial activity. Triphala, an Ayurvedic formula composed of three different plants: *Terminalia chebula* Retz., *Terminalia bellirica* (Gaertn.) Roxb. (Combretaceae), and *Phyllanthus emblica* L. (Phyllanthaceae), is used widely for various microbial infections. Various extraction techniques were applied in the extraction of the biologically active constituents of Triphala in order to compare their efficiency. Microwave-assisted extraction (MAE) was shown to be the most efficient method based on yield, extraction time, and selectivity. The Triphala hydroalcoholic extract (TAE) has been chemically characterized with spectroscopic and chromatographic techniques. Triphala hydroalcoholic extract was evaluated alone or with carvacrol. Different drug formulations including cream and nanoemulsion hydrogel were prepared to assess the antimicrobial activity against selected microorganism strains including Gram-positive and Gram-negative bacteria and fungi. We used a lipophilic oil of carvacrol (5 mg/mL) and a hydrophilic TAE (5 mg/mL) ingredient in a dosage form. Two solutions were created: hydrogel containing nanoemulsion as a lipophilic vector dispersed in the gel as a hydrophilic vehicle and a cream formulation, an oil-in-water emulsion. In both cases, the concentration was 250 mg of active ingredient in 50 mL of final formulation. The formulas developed were stable from a physical and chemical perspective. In the nanoemulsion hydrogel, the oil droplet size ranged from 124 to 129 nm, with low polydispersity index (PdI) 0.132 ± 0.013 and negative zeta potential −46.4 ± 4.3 mV. For the cream, the consistency factor (cetyl alcohol and white wax) induced immobilization of the matrix structure and the stability. Triphala hydroalcoholic extract in drug nanoformulation illustrated might be an adjuvant antimicrobial agent for treating various microbial infections.

## 1. Introduction

Microbial resistance, caused by irrational use of antibiotics, is a major issue in the treatment of infectious diseases. A new strategy to manage microbial resistance is the use of natural derivatives in pure form or in combination with antibiotics or chemotherapeutic agents which, in many cases, create a synergistic effect. The discovery of new sources of natural antimicrobial agents requires the systematic screening of a large number of plants; however, ethnopharmacological data can reduce the time to discover the antimicrobials. Triphala is an Ayurvedic formula used in India historically and currently. It is composed of three plants including *Terminalia chebula* Retz., *Terminalia bellirica* (Gaertn.) Roxb. (Combretaceae), and *Phyllanthus emblica* L. (Phyllanthaceae). Triphala has traditionally been used to treat microbial infections, such as wounds, ulcers, and dental caries [1], and has been subjected to many studies to evaluate its potency as a wound healer and antimicrobial agent. Methanolic extract of Triphala is rich in catechins (catechin, epicatechin, and epigallocatechin gallate). Kumar et al. [2] incorporated a Triphala extract in a collagen sponge to use it as an antimicrobial and wound healing agent, reporting a significant wound closure rate and a significant reduction in the bacterial counts of the infected wounds. Other studies reported the effectiveness of the Triphala extract against a broad spectrum of microorganisms isolated from patients with HIV infection, specifically *Pseudomonas aeruginosa*, *Shigella sonnei*, *Shigella flexneri*, *Vibrio cholera*, *Escherichia coli*, *Enterococcus faecalis*, and *Staphylococcus aureus* [3].

Patients with severe burns and devitalized tissue are frequently immunocompromised. They are highly susceptible to nosocomial infections due to pathogenic bacterial strains including *Pseudomonas aeruginosa*, which could increase the mortality rate [4]. In practice, all known mechanisms of antimicrobial resistance are presented as multiresistant mechanism phenotypes. These include lower outer membrane permeability and active efflux as well as enzymatic inactivation of antibiotics attributed to *β*-lactamases [5, 6]. Since the antimicrobial therapeutic options for multidrug resistant microorganism- (MDRPA-) mediated infections are limited, the medical community is exploring all possibilities to develop novel drugs or therapeutic strategies to manage such infections. Plants are an important source of small molecules, with many exhibiting antimicrobial activity [7–9]. The advances of separation techniques allowed the isolation of many antimicrobial agents from plant origin. Many studies in the field of microbiology and chemotherapy proposed a combination of classic antibiotics with natural antimicrobial agents to manage microbial resistance. As an example, the antimicrobial activity of antibiotics and antimicrobial agents can be enhanced by the combination with natural phytochemicals, especially with essential oil components such as carvacrol or cinnamon oil [10–12].

The aim of this work was to investigate the antimicrobial effect of TAE, extract fractions (**TRIT 1-8**), and some formulations of TAE with natural antimicrobial compound such as carvacrol on different microorganisms.

## 2. Materials and Methods

### 2.1. Plant Material

The authentic sample of Triphala (named TRIT) was purchased from commercial sources: Tausendkraut Naturprodukte, commercial company of natural products (Haupstrasse 68, 69429 Waldbrunn, Germany).

### 2.2. Chemicals and Instruments

#### 2.2.1. Chemicals

Solvents, methanol-d_4_ (99.95%), Ce(SO_4_)_2_, silica gel for TLC aluminum plates (70–220 mesh), carvacrol, propylene glycol, and triethylamine were all purchased from Merck (Sigma-Aldrich). Labrafil® M 1944 CS and Labrafac® WL 1349 (mixture of capric and caprylic acid triglycerides) were obtained from Gattefossé S.A., Saint Priest, France. Nonionic surfactant (Kolliphor® ELP) was obtained from BASF (Ludwigshafen, Germany). Kolliphor® ELP, cetyl alcohol, sodium lauryl sulfate, and white wax were obtained from Cooper, France.

#### 2.2.2. General Experimental Procedures

UV spectra were recorded on a PerkinElmer Lambda spectrophotometer. The NMR experiments were performed on a Bruker DRX-600 spectrometer at 300 K. The mass spectrometry analyses were performed with a Q-TOF premier spectrometer (Waters, Milford, MA, USA), coupled with an Alliance HPLC module (Waters, Milford, MA, USA). TLC was performed on precoated Kieselgel 60 F_254_ plates (Merck, Darmstadt, Germany). Column chromatography was performed over Sephadex LH-20 (Pharmacia); reversed-phase (RP) HPLC separations were conducted on a Shimadzu LC-8A series pumping system equipped with a Shimadzu RID10 A refractive index detector and a Shimadzu injector, using a Bondapak C_18_ column (30 cm × 7.8 mm, 10 *µ*m, Waters, Milford, MA, USA). A Metrohm 827 pH meter, a two-channel laboratory pH measuring instrument for measuring pH/mV and temperature, was used for the pH measurement. The UE was carried out using a 320 W Ultrasonic bath (Branson 2510E-MTH, Bransonic®). The sample was placed in an Erlenmayer flask with the corresponding amount of solvent and was treated with ultrasound at 25°C for a given duration (Table 1). MAE was performed using a multimodal household microwave oven Silvercrest SMW 700 A1 at 700 W and a 100 mL flask exposed to microwave irradiation (irradiation cycle: 10 s power on, followed by 10 s power off) for a given duration (Table 1) [13–15].

### 2.3. Fractionation of Active Ingredients from the Ayurvedic Formula

Triphala was extracted with ethanol (70%) by using different fractionation techniques to evaluate the best fractionation methods for Tausendkraut Bio Triphala (TRIT). Various techniques including ultrasound extraction (UE), microwave-assisted extraction (MAE), and maceration with gentle agitation have been used for the fractionation of the Triphala to shorten the fractionation time, decrease the solvent consumption, increase the fractionation yield, and enhance the quality of the fractions.

Part of Triphala hydroalcoholic extract (TAE) (10 g), obtained by MAE, was partitioned between *n*-BuOH and H_2_O to create a *n*-BuOH residue (5.1 g) with a yield of 51%. A portion of the *n*-BuOH fraction (2.5 g) was chromatographed over a Sephadex LH-20 column (100 cm × 5 cm) with MeOH as the eluent. A total of 80 fractions were collected (10 mL each). These fractions were combined according to the TLC analysis (silica 60 F254 gel-coated glass sheets with CHCl_3_–MeOH (9 : 1), CHCl_3_–MeOH–H_2_O (40 : 9:1), *n*-BuOH–AcOH–H_2_O (60 : 15 : 25), and *n*-BuOH–AcOH–H_2_O (40 : 30 : 30)). These fractions were detected by spraying with Ce(SO_4_)_2_/H_2_SO_4_ solution and grouped into 8 fractions (named **TRIT 1**–**TRIT 8)**. The active fractions **TRIT 6** (90.4 mg), **TRIT 7** (96.4 mg), and **TRIT 8** (231.2 mg) were purified by RP-HPLC using MeOH–H_2_O (3 : 7) to produce the compounds phyllemblin (69,3 mg) from **TRIT 6**; gallic acid (22,3 mg), cinnamic acid (35,5 mg), and 1,3,6-tri-*O*-galloyl-*β*-D-glucose (19,4 mg) from **TRIT 7;** and 1,2,3,6-tetra-*O*-galloyl-*β*-D-glucose (55,3 mg), 1,2,3,4,6-penta-*O*-galloyl-*β*-D-glucose (47,2 mg), p-coumaric acid (57,3 mg), and chebulinic acid (28,4 mg) from **TRIT 8.**

### 2.4. Microbial Strains

Different bacterial strains were used to determine the antibacterial activities of TAE; *Enterococcus faecalis, Staphylococcus aureus, Staphylococcus aureus* (MRSA), *Pseudomonas aeruginosa, Klebsiella pneumoniae* (ESBL), *Escherichia coli, Proteus mirabilis*, and *Acinetobacter baumannii* were isolated from patients at Al-Noor Specialist Hospital, Makkah, Saudi Arabia. The identification and susceptibility patterns of all clinical isolates were performed using the VITEK 2 compact system. The ATCC bacterial strains were divided in two groups. The Gram-positive bacteria group included *E. faecalis* (VRE) ATCC 29212, *E. faecalis* ATCC 35218, *E. faecalis* ATCC 51299, *S. aureus* (MRSA) ATCC 43300, and *S. epidermidis* ATCC 12228. The Gram-negative bacteria group included *Proteus mirabilis* ATCC 43071*, K. pneumonia* (CRE) ATCC 700603, *S. typhimurium* (ESBL) ATCC 14028, *K. pneumonia* ATCC 1705, *Shigella sonnei* ATCC 25931, and *P. mirabilis* ATCC 43071. The bacterial strains were subcultured on Muller Hinton agar plates and incubated at 37°C overnight and a single colony was obtained using a sterile loop and inoculated in 3 ml of Muller Hinton broth to form a homogenous suspension of the test organism. The suspension was standardized to provide 0.5 McFarland = 1 − 2*x*CFU/mL using calibrated VITEK 2 DENSICHEK (bioMérieux, Inc.).

In addition, different bacterial, yeast, and mould strains recommended by the European Pharmacopoeia have been used to determine the antimicrobial activities of the TAE-carvacrol drug formulations, specifically the mould strain *Aspergillus niger* (ATCC 16404), the yeast strain *Candida albicans* (ATCC 10231), and bacterial strains *S. aureus* (ATCC 43300), *P. aeruginosa* (ATCC 9027), and *E. coli* (ATCC 35218). Also, other bacterial strains such as *Staphylococcus capitis*, *Staphylococcus epidermidis*, and *Enterococcus faecalis* and spores such as *Bacillus cereus*, *Bacillus subtilis*, and *Bacillus sphaericus* were tested. Supplemented with 20% glycerol, before their use, the bacteria were kept frozen at −80°C in brain heart infusion broth (bioMérieux, Marcy l'Etoile, France).

At 37°C, bacterial strains were grown on trypticase soy agar (TSA) (bioMéieux, Marcy l'Etoile, France). At 30°C, mould and yeast strains were grown on trypticase soy agar (bioMérieux, Marcy l'Etoile, France). At 30°C, by suspending overnight the strains, these ones were grown in a Mueller Hinton broth (bioMérieux, Marcy l'Etoile, France). In order to prepare 1–3 x 10^8^ colony forming units (CFU)/mL, the cultures were prepared and adjusted to an OD620 of 0.15–0.16. *A. niger* (ATCC 16404) (5.4 × 10^6^ CFU/tablet) and *C. albicans* (ATCC 10231) (6.7 × 10^7^ CFU/tablet) were provided by AES Laboratoire (Combourg, France). The determination of MIC for *A. niger* and *C. albicans* has been realised in Sabouraud medium broth (bioMérieux, Marcy l'Etoile, France).

*B. subtilis* spores (10^6^ spores/tablet) were provided by AES Laboratoire, Combourg, France. *B. cereus* and *B. sphaericus* spores were isolated from growth culture on a specific medium (meat fraction containing 2.4 g; peptone 2.4 g; NaCl 1.2 g; MnSO_4_·4 H_2_O 0.03 g; KH_2_PO_4_ 0.25 g; agar 13g; water 1000 mL). In particular, *B. cereus* and *B. sphaericus* spores were obtained after 48 h culture at 37°C on a slant of specific medium. This one was shaken for 15 s to be flooded after with 2 mL of sterile water. Then, the water was transferred to a sterile tube and heated for 15 min at 80°C. Serial 10^−1^ dilutions of the spore solutions were enumerated by the plating method on TSA medium.

### 2.5. Antimicrobial Activities of TAE and TRIT Fractions

#### 2.5.1. Microdilution of Broth Assay

The microdilution method, using 96-well microtiter plates according to the Clinical and Laboratory Standards Institute (CLSI) guideline [16], was conducted to evaluate the antibacterial activity. Performance standards for antimicrobial susceptibility testing were based on the 18^th^ informational supplement of CLSI document Wayne (PA Clinical Laboratories Standards Institute, pp. 46–52). Briefly, *P. aeruginosa* ATCC 27853 was grown overnight in Mueller Hinton broth (MHB) medium at 37°C. Cultures were then diluted to approximately 10^5^ CFU/mL in fresh MHB medium, and 200 *μ*L was used to inoculate flat-bottom 96-well polystyrene microtiter plates.

#### 2.5.2. Determination of the Minimum Inhibitory Concentration (MIC) and Minimum Bactericidal Concentrations (MBCs) of TAE

Stock solutions of TAE (ethanol 70% obtained with microwave-assisted fractionation) were prepared by solubilization of 50 mg of fraction in 1 mL of distilled water. The broth microdilution method was used with sterile 96-well microtiter plates for the determination of the MIC and MBCs of the TAE. Basically, the first column in microtiter plates contained 200 *µ*L of the plant fraction at the highest concentration (50 mg/mL) and 100 *µ*L Mueller Hinton broth in other wells. TAE was serially diluted by transferring 100 *µ*L to the next well to produce final concentrations of 0.32, 0.75, 1.5, 3, 6, 12, 25, and 50 mg/mL. Mueller Hinton broth (100 *µ*L containing the bacteria 0.5 McFarland) was added to each well containing 100 *µ*L plant fraction in the dilution series as well as a positive control well and mixed. Sterilized Mueller Hinton broth alone was used as negative control, and bacterial broth with dimethyl sulfoxide (DMSO) only was used as control. The microdilution plates were incubated at 37°C overnight. The MIC was determined by selecting the lowest concentration of TAE that completely inhibited the growth of the organism and compared with the growth control (Table 2). Wells with no visible growth in MIC were subcultured using 10 *μ*L of the selected wells and placed on Muller Hinton agar plates. The MBC was determined by taking 10 *μ*L of the selected column and placing it on the Mueller Hinton agar plates as well. All plates were incubated for 24 h at 37°C and the colony forming units (CFUs) were counted. MIC was determined by selecting the lowest concentration of TAE that completely inhibited the visible growth of a microorganism after overnight incubation in the well. The MBC was defined as the lowest concentration of the TAE that prevents any growth of an organism after being subcultured on the Mueller Hinton agar plate.

#### 2.5.3. Disc Diffusion Test

In brief, a Whatman filter paper no. 1 was used to prepare discs approximately 6 mm in diameter, which were sterilized in a hot air oven according to the Clinical and Laboratory Standards Institute (CLSI) guideline [17] and spotted with 0.2 mg TRIT fractions. DMSO was used as the control to ensure the vitality of the tested microorganisms and the accuracy of the positive and negative control. Carvacrol was used as positive control (data not shown). The resulting filters were then placed on a Mueller Hinton agar (MHA) plate on which a bacterial suspension (OD600 nm = 0.5) of the quality control strain *P. aeruginosa* ATCC 27853 was spread. The plates were incubated at 37°C for 24 h and the diameter of the inhibition zones of growth produced by each spot was measured in millimetres.

### 2.6. Formulation of TAE-Carvacrol Cream

The oil phase used in the preparation of oil-in-water (O/W) cream was cetylic alcohol and white wax. Carvacrol was beforehand solubilized in propyleneglycol and after in melted cetylic alcohol and white wax. Oil phase is mixed with sodium laurylsufate for 1 minute at 70°C and homogenized. The aqueous phase containing TAE was added to the oil phase, homogenized, and left to cool to room temperature to form an emulsion consisting of small oil droplets dispersed in water. The cream containing 0.5% TAE and 0.5% carvacrol was prepared by adding 8% of cetylic alcohol, 12% of white wax, 12% propyleneglycol, 1% sodium laurylsufate, and 66.04% deionized water.

### 2.7. Formulation of TAE-Carvacrol Nanoemulsion Hydrogel

The oil phase used in the preparation of nanoemulsions was Labrafac® WL 1349. Carvacrol was beforehand solubilized in oil during 2 hours at 40°C. Oil phase is mixed with Kolliphor® ELP for 1 minute at 80°C and homogenized with Vortex. Water containing solubilized TAE was suddenly added, and the nanoemulsions were immediately formed, sizing at 126 nm. The gelling polymer (Carbopol 940) was then mixed with the nanoemulsion and gently stirred overnight until hydration of the polymer. The final step is the addition of triethylamine to neutralize the pH and induce the gelification. The nanoemulsion hydrogel containing 0.5% TAE and 0.5% carvacrol was prepared by adding 14.6% of Labrafac®WL1349, 14.6% of Kolliphor®ELP, 0.5% Carbopol 940, and 69.34% deionized water.

### 2.8. Dynamic Light Scattering

Size distributions and polydispersity indices (PDI) were measured by dynamic light scattering (DLS), with a NanoZS Malvern apparatus (Malvern, Orsay, France). The helium/neon laser, 4 mW, was operated at 633 nm, with the scatter angle fixed at 173°, and the temperature was maintained at 25°C. DLS data were analyzed using a cumulant-based method.

### 2.9. Formulations' Stability

Information on the stability of dosage forms under defined storage conditions is an integral part of the systematic approach to stability evaluation. Stress testing helps to determine the intrinsic stability characteristics of a molecule by establishing degradation pathways to identify the likely degradation products and the destabilization of formulations like emulsions, cream, or gel. The gel form was stored at room temperature and protected from light for 2 months. The nanoemulsion hydrogel was monitored for any physical instabilities during stress testing (indicated by phase separations, drug precipitations, or color changes) to exclude this nanoformulation from further investigation and characterization.

### 2.10. Antimicrobial Activities of TAE Drug Formulations

#### 2.10.1. Determination of the Minimum Bactericidal Concentration (MBC) and the Minimum Inhibitory Concentration (MIC) of TAE-Carvacrol Drug Formulations

The MBC and MIC were determined in aseptic conditions to avoid microbial contamination. The experimental determination did not affect any microorganisms used in the test. As described above, the MIC was determined in a Mueller Hinton broth (bioMérieux, Marcy l'Etoile, France).

Nanoemulsioned hydrogels containing TAE and carvacrol (same amounts than those used in the formulation of the cream) were sterilized before use by filtration using 0.45 *μ*m pore size filters (Dutscher, Brumath, France). Firstly, a series of test tubes containing different concentrations in descending order of nanoemulsions was prepared in a Mueller Hinton broth. These series were inoculated with the same amount of spores or bacteria (10^6^ CFU/ml). As a control for organism viability, a broth did not contain any antimicrobial agent. The MIC value (mg/mL) was determined from the test tube with the highest concentration in which no microbial growth was observed after 18–24 h of incubation at 37°C. Secondly, 100 *μ*L of the samples was taken from each test tube with no growth. The enumerations were done by the plating method on Sabouraud agar or Mueller Hinton on the serial 10^−1^ dilutions of the samples. The number of surviving microorganisms (*C. albicans* and *A. niger*) was counted and compared to the standard range (10^6^ CFU/mL) after 24 h incubation at 37°C or 48 h at 30°C. The MBC value (mg/mL) was determined by the concentration of the sample tube in which surviving microorganisms were equivalent to 0.01%. The antibacterial activity was determined by the ratio MBC/MIC as follows:

  MBC/MIC > 4 refers to a bacteriostatic, fungistatic, or sporostatic activity
  MBC/MIC < or = 4 refers to a bactericidal, fungistatic, or sporicidal activity

#### 2.10.2. Test for Efficacy of Antimicrobial Preservation

The efficacy of antimicrobial activity for liquid formulations was determined following the test described in the 10^th^ edition of the European Pharmacopoeia. Challenge testing was performed using microorganisms selected from those listed in Section 5.1.3 of the 10^th^ edition of the EP (*E. coli* (ATCC 8739), *P. aeruginosa* (ATCC 9027), *S. aureus* (ATCC 6538), *A. niger* (ATCC 16404), and *C. albicans* (ATCC 10231)). The samples to be examined were inoculated with a suspension of one of the test organisms in order to give an inoculum at 10^6^ CFU/g.

The inoculated product (30 g) was maintained at 20°C–25°C in the dark. One gram of the inoculated product was removed at 0, 14, and 28 days. To each aliquot, 9 mL of a neutralizing solution was added (lecithin, 3 g; polysorbate 80, 30 g; sodium chloride, 1 g; histidine monohydrochloride, 3.6 g; sodium phosphate dibasic, 4.4 g; potassium phosphate dibasic, 7.2 g; water, 1000 mL). After being allowed to neutralize for 20 min at room temperature, broths were diluted until 10^−5^ into the neutralizing solution and filtered through 0.45 *μ*m filters (Millipore, Molsheim, France). Neutralizing solution was used to rinse five times each membrane filter with 10 mL. In a last step, each membrane was plated on an appropriate agar plate. Bacterial plates were incubated at 30°C for 24 h. Mould and yeast plates were incubated 48 h at 30°C and the CFUs were counted. The control preparations were similarly sampled at 0 h to determine the viable counts of the cultures used and to confirm the ability of the unpreserved formulation to support the viability and/or the microbial growth and also the effectiveness of the neutralizing medium for the inoculum recovery [18].

### 2.11. Cell Culture and Viability

HeLa (human cervix epitheloid carcinoma) cells were obtained from Cell Bank in GMP-IST (Genova, Italy). The HeLa cells were cultured in Dulbecco's modified Eagle medium (DMEM), supplemented with 10% (v/v) FBS, 2 mM L-glutamine, and antibiotics (100 U/mL penicillin and 100 *µ*gmL^−1^ streptomycin) purchased from Invitrogen (Carslbad, CA, USA). Cells were grown at 37°C in a humidified atmosphere containing 5% CO_2_. Human peripheral blood mononuclear cells (PBMC) were isolated from buffy coats of healthy donors (kindly provided by the Blood Center of the Hospital of Battipaglia, Salerno, Italy) by using a standard Ficoll-Paque gradient. Freshly isolated PBMC contained 90.6 ± 1.2% live cells as assessed by the manual trypan blue exclusion method. Resting PBMC and PBMC induced to proliferate by phytohemagglutinin (PHA) (10 *μ*g/ml) were used to evaluate TAE and active fractions' (**TRIT 4**–**TRIT 8**) cytotoxic and cytostatic effects, respectively. To ensure logarithmic growth, cells were subcultured every 2 days. In all experiments, the final concentration of DMSO did not exceed 0.15% (v/v). Cells were seeded in 96-well plates and incubated for the established times in the absence (vehicle only) and in the presence of different concentrations of samples (0.32, 0.75, 1.5, and 3 mg/mL). The day before treatments, cells were seeded at a cell density of 1 × 10^4^ cells/well. The number of viable cells was quantified by MTT assay by using etoposide, E1383, synthetic, ≥98%, Sigma-Aldrich, as positive control [19]. Absorption at 550 nm for each well was assessed using a microplate reader (LabSystems). In some experiments, cell viability was also checked by trypan blue exclusion assay using a Bürker counting chamber. IC_50_ values were calculated from cell viability dose-response curves and defined as the concentration resulting in 50% inhibition in cell survival as compared to controls [20].

## 3. Results

### 3.1. Extracts and Components from Triphala

Different parameters of the extracts obtained with the different extraction techniques were measured. The values of pH, time, and dry residue were collected for all. The values are shown in Table 1. The extracts were primarily analyzed by UV-Vis and NMR spectroscopy to evaluate the extraction of compounds present within the extracts obtained with different extraction techniques. By comparing the spectra obtained for the extract by the MAE method compared to those obtained by maceration and UE, it was found that the first method had a higher content in molecules (see Figures S1–S5 in the Supplementary Material for the NMR spectra of the TAE extracts).

All data revealed that the MAE method provided high extraction yield, requiring short timeframes and less labour (Table 1), so it was chosen as the method for Triphala extraction.

**Figure 1:**
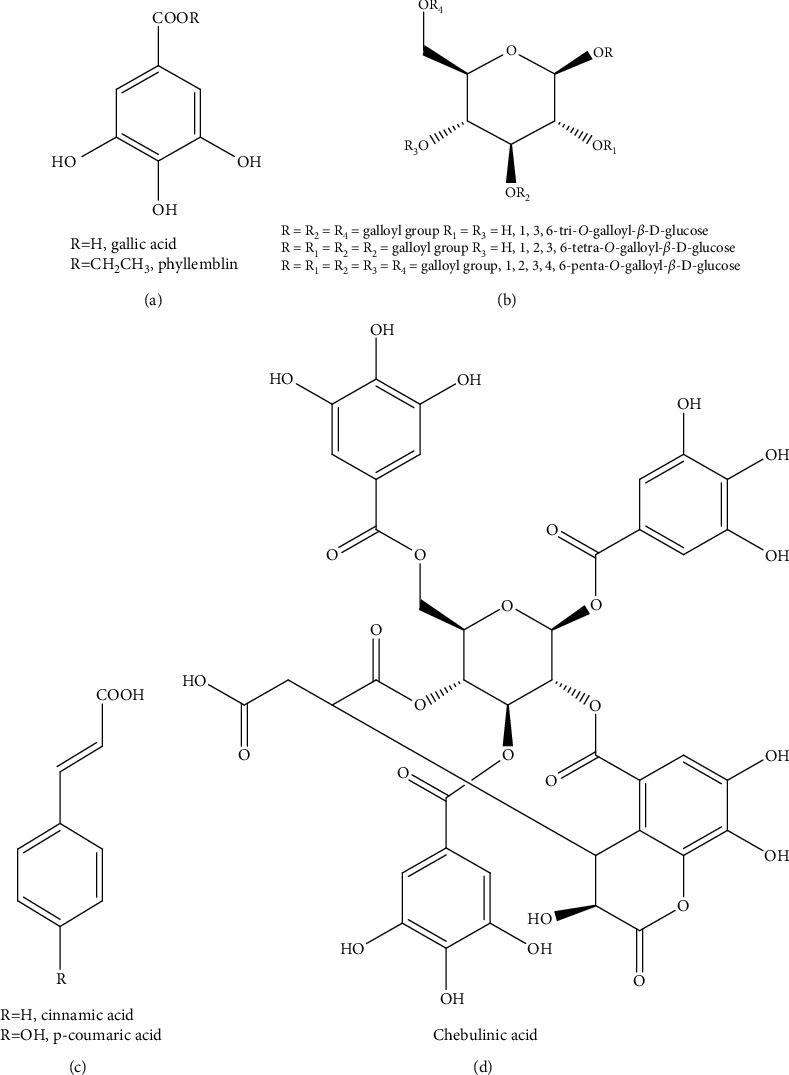
Structures of the isolated compounds.

The active fractions **TRIT 6**, **TRIT 7**, and **TRIT 8** from Triphala hydroalcoholic extract, obtained by MAE, were purified with RP-HPLC using MeOH–H_2_O (3 : 7) to produce compounds of phyllemblin (C_9_H_10_O_5_ showed an [M-H]^−^ ion at *m/z* 197.0531) [21] from **TRIT 6**; gallic acid (C_7_H_6_O_5_ showed an [M-H]^−^ ion at *m/z* 169.0217), cinnamic acid (C_9_H_8_O_2_ showed an [M-H]^−^ ion at *m/z* 147.0525), and 1,3,6-tri-*O*-galloyl-*β*-D-glucose (C_27_H_24_O_18_ showed an [M-H]^−^ ion at *m/z* 635.0969) [22, 23] from **TRIT 7**; and 1,2,3,6-tetra-*O*-galloyl-*β*-glucose (C_34_H_28_O_22_ showed an [M-H]^−^ ion at *m/z* 787.1075), 1,2,3,4,6-penta-*O*-galloyl-*β*-D-glucose (C_41_H_32_O_26_ showed an [M-H]^−^ ion at *m/z* 939.1187), p-coumaric acid (C_9_H_8_O_3_ showed an [M-H]^−^ ion at *m/z* 163.0476) [23], and chebulinic acid (C_41_H_32_O_27_ showed an [M-H]^−^ ion at *m/z* 955.1134) [24] from **TRIT 8** (Figure 1), molecules known in the literature for their antimicrobial activity [25–31].

The structure of each compound was determined by NMR (see Figures S6–S13 in the Supplementary Material for the ^1^H NMR spectra of the isolated compounds).

### 3.2. Antibacterial Activity of TAE against Bacterial Strains

TAE was examined against several Gram-positive and Gram-negative ATCC bacterial strains (Table 2). *Proteus mirabilis* ATCC 43071 and *Escherichia coli* ATCC 35218 were the most resistant strains with no antibacterial activity at 50 mg/mL TAE. *Staphylococcus epidermidis* ATCC 12228 was the most sensitive strain; its growth was inhibited at a concentration of 3 mg/mL and completely eradicated at 6 mg/mL. TAE illustrated antibacterial activity against *Acinetobacter baumannii* ATCC 19605, *Salmonella typhimurium* ATCC 14028, and *Staphylococcus aureus* ATCC 43300 strains. The same inhibition and eradication manner was measured for all at 12 and 25 mg/mL, respectively. In addition, TAE slightly affected the antibacterial activity against *Shigella sonnei* ATCC 25931, *Klebsiella pneumoniae* (CRE) ATCC 1705, *Klebsiella pneumoniae* (ESBL) ATCC 700603, *Staphylococcus aureus* (MRSA) ATCC 43300, *Enterococcus faecalis* ATCC 29212, and *Enterococcus faecalis* (VRE) ATCC 51299. The MIC value was 25 mg/mL, and the eradication concentration was 50 mg/mL for all (Table 2).

The antibacterial activity of TAE was also investigated with bacterial clinical isolates provided from Al-Noor Specialist Hospital, Makkah, Saudi Arabia, during the Muslim pilgrimage and was found active against *Pseudomonas aeruginosa*, *Klebsiella pneumoniae* (ESBL), *Escherichia coli*, and *Proteus mirabilis* clinical isolates. Growth inhibition effects at 12 mg/mL and eradication at 25 mg/mL were observed. TAE had relative antibacterial activity against other bacterial clinical strains such as *Staphylococcus aureus*, *Enterococcus faecalis*, and *Acinetobacter baumannii* but was inactive against *Staphylococcus aureus* (MRSA) (Table 2).

### 3.3. Antibacterial Activity of **TRIT 1–TRIT 8** against *P. aeruginosa* ATCC 27853

**Figure 2:**
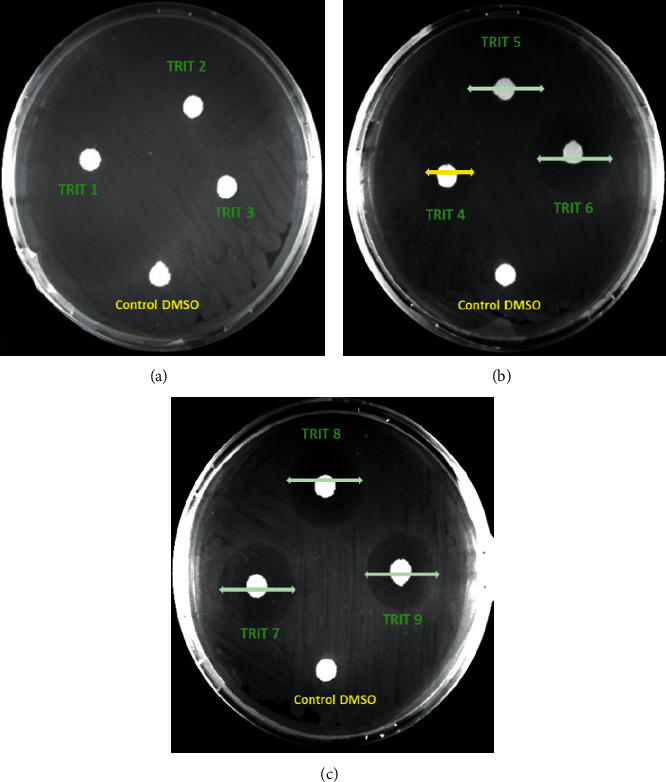
Disc diffusion test of TRIT 1–TRIT 8 against *P. aeruginosa* ATCC 27853; TRIT 9 was the total Triphala hydroalcoholic extract.

**Figure 3:**
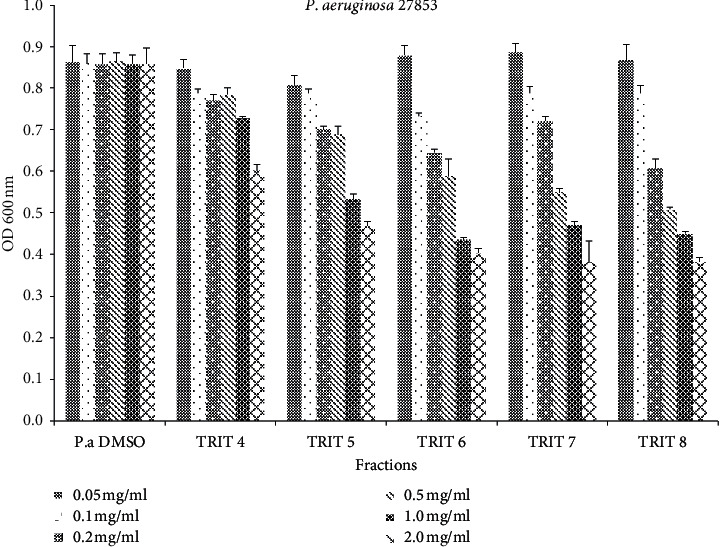
Microdilution broth assay of TRIT 4–TRIT 8 against *P. aeruginosa* ATCC 27853.

The results *indicated* that only fractions **TRIT 5, TRIT 6, TRIT 7**, and **TRIT 8** exhibited antibacterial activity in a concentration-dependent manner against *P. aeruginosa* ATCC 27853 (ampicillin resistant). Both **TRIT 4-8** and TAE extract (TRIT 9) inhibited *P. aeruginosa* growth in an amount of 2 mg, which is observed as the presence of a halo of inhibition around the disk (Figure 2). In particular, **TRIT 6, TRIT 7**, and **TRIT 8** inhibited 50% of the bacterial growth (MIC_50_) at a concentration of 1.0 mg/ml. **TRIT 5** exhibited a MIC_50_ at 2.0 mg/mL, and **TRIT 4** at 2.0 mg/mL inhibited only 30% of the bacterial growth (MIC_30_) (Figures 2 and 3).

### 3.4. Microbiological Properties of Nanoemulsion Hydrogel Containing TAE and Carvacrol

The observed MICs and MBCs values obtained for nanoemulsion hydrogel containing TAE and carvacrol varied between microorganisms (Table 2) in a range of 20 mg/100 mL to 75 mg/100 mL and of 75 mg/100 mL to 200 mg/100 mL, respectively. The determination of the MIC allows to conclude that *Bacillus* spores were the highest resistant microorganism with a MIC of 50–75 mg/mL, whereas the most sensitive strains were *A. niger*, *E. coli*, and *S. capitis*. The Gram-negative bacteria *E. coli* and *P. aeruginosa* showed higher values of 200 mg/100 mL. Nanoemulsions containing TAE and carvacrol were fungistatic or bacteriostatic for all the tested strains except *S. aureus* and Gram-negative bacteria for which a bactericidal activity was shown. The cream formulation was not active at the same concentrations used for the nanoemulsion hydrogel, and for this reason these data were not reported in Table 2. This was probably due to slower release rate of the active ingredients from the conventional formulation in comparison to the nanoformulation.

## 4. Discussion

The obtained extract was used to evaluate the microbial test against Gram-positive and Gram-negative bacteria obtained from clinical isolates from Al-Nour Specialist Hospital, Makkah, Saudi Arabia, including *Enterococcus faecalis*, *S. aureus*, *S. aureus* (MRSA), *P. aeruginosa*, *K. pneumonia* (ESBL), *E. coli*, *P. mirabilis*, *A. baumannii*, and 13 ATCC bacterial strains. Five of these strains were related to Gram-positive bacteria: *E. faecalis* (VRE) ATCC 29212, *E. faecalis* ATCC 35218, *S. aureus* ATCC 51299*, S. aureus* (MRSA) ATCC 43300, and S*. epidermidis* ATCC 43300. Eight strains were related to Gram-negative bacteria: *E. coli* ATCC 43071, *Klebsiella pneumoniae* (CRE) ATCC 700603, *Klebsiella pneumoniae* (ESBL) ATCC 14028, *Acinetobacter baumannii* ATCC 1705, *Salmonella typhimurium* ATCC 12228, *Shigella sonnei* ATCC 25931, and *Proteus mirabilis* ATCC 43071. In total, 17 stains were sensitive to TAE, and the MIC ranged between 3 mg/mL and 25 mg/mL of the crude fraction. The most sensitive strain was *Staphylococcus epidermidis* ATCC 12228 with a MIC = 3 mg/mL, followed by *Staphylococcus aureus* ATCC 43300, *Salmonella typhimurium* ATCC 14028, and *Acinetobacter baumannii* ATCC 19605. Some were clinical isolates including *Pseudomonas aeruginosa* clinical isolate*, Klebsiella pneumoniae* (ESBL) *Escherichia coli*, and *Proteus mirabilis* with a MIC = 12 mg/mL. Less sensitive strains included *Enterococcus faecalis* ATCC 29212, *Enterococcus faecalis* (VRE) ATCC 51299, *Staphylococcus aureus* (MRSA) ATCC 43300, *Klebsiella pneumoniae* (ESBL) ATCC 700603, *Klebsiella pneumoniae* (CRE) ATCC 1705, and *Shigella sonnei* ATCC 25931. For the clinical isolates *Enterococcus faecalis*, *Staphylococcus aureus*, and *Acinetobacter baumannii,* MIC = 25 mg/mL was required. Nonsensitive strains at 50 mg/mL were *Proteus mirabilis* ATCC 43071, *Escherichia coli* ATCC 35218, and *Staphylococcus aureus* (MRSA) derived from the clinical isolates.

The hydroalcoholic extract of Triphala was fractioned through gel filtration using Sephadex LH-20, yielding simple phenylpropanoids (p-coumaric acid and cinnamic acid) and polyphenolic derivatives. The antimicrobial effect of the semipurified fractions was slightly improved compared to the crude extract. However, we decided to use the hydroalcoholic extract of Triphala for further combination with natural antimicrobial agents. We combined the extract with carvacrol, known for its antimicrobial activity against a broad range of microorganisms including *Pseudomonas aeruginosa* [32–34]. The combination of carvacrol with Triphala 70% ethanol extract (TAE) exhibited improvement in the antimicrobial activity against *Pseudomonas aeruginosa*.

We used a lipophilic oil of carvacrol (5 mg/mL) and a hydrophilic TAE (5 mg/mL) ingredient in a dosage form. Two solutions were created: hydrogel containing nanoemulsions as a lipophilic vector dispersed in the gel as a hydrophilic vehicle and a cream formulation, i.e., an oil-in-water emulsion (classical pharmaceutical formulation). In both cases, the concentration was 250 mg of active ingredient in 50 mL of final formulation. The global visual aspect of this dispersed system has been totally unchanged. No phase separation as well as heterogeneity, odor or color changes, or signs of microorganisms or fungus development was observed.

The formulas developed were stable from a physical and chemical perspective, due to, in the case of the gel, the intrinsic stability of the nanoemulsion hydrogel, coupled with the immobilization due to the gel itself. The oil droplet size ranged from 124 to 129 nm, with low polydispersity index (PdI) 0.132 ± 0.013 and negative zeta potential −46.4 ± 4.3 mV. For the cream, the consistency factor (cetyl alcohol and white wax) induced immobilization of the matrix structure and the stability.

Different bacterial strains described in the European Pharmacopoeia have been used to study the antibacterial activity of the TAE formulations. Easily dispersed by skin scales, coagulase-negative *Staphylococci* such as *S. capitis* and *S. epidermidis* belong to the microflora of mucous membranes and human skin. In immunocompetent adults, they are considered opportunistic pathogens and are mostly associated with foreign body infections, osteomyelitis, endocarditis, and surgical site. Also, one can mention that *S. epidermidis* is a major cause of nosocomial bacteraemia. Several virulence factors can be expressed such as the ability to accumulate and to adhere as a biofilm on surfaces such as transcutaneous catheters, skin, and prosthetic devices. *E. faecalis* is the main species found in human faeces. *Enterococci*, frequently isolated in various foods, soil, vegetables, and plants, are naturally members of the warm-blooded animal and human intestinal flora. Due to their high concentrations in faeces and their long survival period in the environment, *Enterococci* have been suggested as a water faecal contamination indicator. *Bacillus* species are able to produce a dormant endospore by sporulation. These spores are highly resistant to environmental stresses, including extremes of ionic strength, heat, toxic chemicals, and desiccation [35], whereas environmental extremes can readily kill vegetative bacteria, and bacterial spores are able to remain viable during sterilization processes. Due to their high resistance to environmental stresses, these spores are employed as biological indicators for monitoring the effectiveness of sterilization processes, such as autoclaving, vaporized hydrogen peroxide treatments, and UV irradiation. Interestingly, nanoemulsion hydrogel containing TAE and carvacrol formulation had a bactericidal effect for both Gram-negative and Gram-positive bacteria except *S. epidermidis,* for which it was only bacteriostatic. Moreover, the cytotoxic activity of the extract and the most active fractions **TRIT 6, 7,** and **8** was evaluated in HeLa cell line and in normal cells (PBMC). All samples showed no cytotoxicity, with 50% cell growth inhibition (IC_50_) values always greater than 2 mg/mL. The purpose of these formulations is to enhance the TAE antimicrobial activity; however, the synergistic effect of combining an antibiotic and TAE in this formulation to improve the antimicrobial activity will be addressed in the future work plan of this project.

## 5. Conclusions

Drugs obtained from natural sources are readily available due to the growth in identification and purification techniques, and as a result, the number of isolated natural constituents is increasing constantly. Currently, the pharmaceutical industry is using natural drugs globally, not only due to the availability and fewer side effects but also due to the economic impact on third world countries with a low income which are dependent on natural remedies. The Triphala formula is used in India historically as well as currently, and it has recently emerged as a potential candidate for the treatment of various ailments. In the current study, we focused on the possibility of using TAE as a natural antimicrobial agent in combination with other natural antimicrobial agents to improve the efficacy synergistically. The novel nanoemulgel of TAE-carvacrol with suitable viscosity was successfully formulated for a future transdermal application. Nanoemulgel was formulated by addition of Carbopol 940 into nanoemulsion which resulted in increase in viscosity. The optimized formulation was compared with conventional cream formulation, and it showed significant higher antimicrobial activity which justifies the nanoemulgel system to be a promising surrogate carrier for delivery of TAE-carvacrol. Of importance, the results of the study can be used in the industrial field to optimize the nanoformula for topical application and to consider encapsulation of conventional antibiotics with TAE to improve the antimicrobial activity.

## Figures and Tables

**Table 1:** Comparison of the extraction yields (percentage of extracted matter from raw Triphala) for the maceration extraction, UE, and MAE^*∗*^.

Extraction type	Triphala/solvent (70% ethanol) (m/V)	pH	Time	Total extract (%)
Maceration	1 : 20	6.36	72 h	39.3 ± 2
UE	1 : 20	6.64	30 min	39.9 ± 1
MAE	1 : 20	6.71	3 × 10 s	54.0 ± 1

^*∗*^Mean values of the three extractions ± SD.

**Table 2:** MIC, MBC, and MFC (minimum fungicidal concentration) of TAE and nanoemulsion hydrogel containing TAE and carvacrol for different microorganism strains.

Microorganism	mg/mL			
	MIC	MBC	MIC/MBC	Activity
TAE				
*Enterococcus faecalis*, ATCC-29212	25	50	<4	Bactericidal
*Enterococcus faecalis (VRE)*, ATCC-51299	25	50	<4	Bactericidal
*Staphylococcus aureus*, ATCC-43300	12	25	<4	Bactericidal
*Staphylococcus aureus (MRSA)*, ATCC-43300	25	50	<4	Bactericidal
*Staphylococcus epidermidis*, ATCC-12228	3	6	<4	Bactericidal
*Salmonella typhimurium*, ATCC-14028	12	25	<4	Bactericidal
*Klebsiella pneumoniae (ESBL)*, ATCC-700603	25	50	<4	Bactericidal
*Klebsiella pneumoniae (CRE)*, ATCC-1705	25	50	<4	Bactericidal
*Acinetobacter baumannii*, ATCC-19605	12	25	<4	Bactericidal
*Shigella sonnei*, ATCC-25931	25	50	<4	Bactericidal
*Proteus mirabilis*, ATCC-43071	NA	NA	—	NA
*Escherichia coli*, ATCC-35218	NA	NA	—	NA
*Pseudomonas aeruginosa*	12	25	<4	Bactericidal
*Klebsiella pneumoniae (ESBL)*	12	25	<4	Bactericidal
*Escherichia coli*	12	25	<4	Bactericidal
*Staphylococcus aureus*	25	50	<4	Bactericidal
*Enterococcus faecalis*	25	50	<4	Bactericidal
*Acinetobacter baumannii*	25	50	<4	Bactericidal
*Staphylococcus aureus (MRSA)*	NA	NA	—	NA
*Proteus mirabilis*	12	25	<4	Bactericidal

TAE and carvacrol nanoemulsion hydrogel				
*Escherichia coli*	36.6	75	<4	Bactericidal
*Staphylococcus aureus*	46.6	150	<4	Bactericidal
*Enterococcus faecalis*	50	200	<4	Bactericidal
*Staphylococcus epidermidis*	40	200	>4	Bacteriostatic
*Staphylococcus capitis*	20	87.5	<4	Bactericidal
*Bacillus cereus spores*	66.6	>200	>4	Sporostatic
*Bacillus sphaericus spores*	58.3	>200	>4	Sporostatic
*Bacillus subtilis spores*	75	>200	>4	Sporostatic

	MIC	MFC	MIC/MFC	Activity
*Candida albicans*	36.6	200	>4	Fungistatic
*Aspergillus niger*	33.3	>200	>4	Fungistatic

NA = no antibacterial activity at the concentration of the extract tested (50 mg/mL).

## Data Availability

The data used to support the findings of this study are available from the corresponding authors upon request.

## Supplementary Materials

Supplementary Materials Figure S1: 1H NMR spectrum of TAE by maceration (CD3OD, 600 MHz). Figure S2: 1H NMR spectrum of TAE by UE (CD3OD, 600 MHz). Figure S3: 1H NMR spectrum of TAE by MAE (CD3OD, 600 MHz). Figure S4: HMBC NMR spectrum of TAE (CD3OD, 600 MHz). Figure S5: HSQC NMR spectrum of TAE (CD3OD, 600 MHz). Figure S6: 1H NMR spectrum of phyllemblin (CD3OD, 600 MHz). Figure S7: 1H NMR spectrum of gallic acid (CD3OD, 600 MHz). Figure S8: 1H NMR spectrum of cinnamic acid (CD3OD, 600 MHz). Figure S9: 1H NMR spectrum of 1,3,6-tri-O-galloyl-*β*-D-glucose (CD3OD, 600 MHz). Figure S10: 1H NMR spectrum of 1,2,3,6-tetra-O-galloyl-*β*-glucose (CD3OD, 600 MHz). Figure S11: 1H NMR spectrum of 1,2,3,4,6-penta-O-galloyl-*β*-D-glucose (CD3OD, 600 MHz). Figure S12: 1H NMR spectrum of p-coumaric acid (CD3OD, 600 MHz). Figure S13: 1H NMR spectrum of chebulinic acid (CD3OD, 600 MHz).
