# Supplementary material for: Evaluation of Antimicrobial Activity of Triphala Constituents and Nanoformulation
Source: Evid Based Complement Alternat Med. 2020 Aug 2;2020:6976973. doi: 10.1155/2020/6976973 (PMC7422007; doi:10.1155/2020/6976973)

Supplementary Material

Evaluation of Antimicrobial Activity of Triphala Constituents and Nanoformulation.

Ziad Omran[1, 9], Ammar Bader[1], Amalia Porta[2], Thierry Vandamme[3], Nicolas Anton[3], Zeyad Alehaideb[4], Hamdi El-Said[5], Hani Faidah[5, 6], Abulrahman Essa[7], Antonio Vassallo[8,*] and Majed Halwani[9, *]

[1] 1College of Pharmacy, Umm Al-Qura University, Makkah, Saudi Arabia
[2] Dipartimento di Farmacia, Università di Salerno, via Giovanni Paolo II 132, 84084 Fisciano (SA), Italy
[3] University of Strasbourg, Faculty of Pharmacy, Strasbourg, France
[4] Department of Medical Genomics, King Abdullah International Medical Research Center, King Saud Bin Abdulaziz University for Health Sciences, Riyadh, Saudi Arabia
[5] Department of Medical Microbiology, Faculty of Medicine, Umm Al-Qura University, Makkah, Saudi Arabia
[6] Al-Noor Specialist Hospital, Makah City, Saudi Arabia
[7] Research Centre, King Fahd Armed Forces Hospitals, Jeddah, Saudi Arabia
[8] Dipartimento di Scienze, Università degli Studi della Basilicata, Italy
[9] King Abdullah International Medical Research Center, King Saud bin Abdulaziz University for Health Sciences, Riyadh, Saudi Arabia

* Equal contributing authors

Correspondence should be addressed to Antonio Vassallo; antonio.vassallo@unibas.it and Majed Halwani; majed.halwani@gmail.com

Contents

Figure S1. ^{1}H NMR spectrum of TAE by maceration (CD_3OD, 600 MHz).

Figure S2. ^{1}H NMR spectrum of TAE by UE (CD_3OD, 600 MHz).

Figure S3. ^{1}H NMR spectrum of TAE by MAE (CD_3OD, 600 MHz).

Figure S4. HMBC NMR spectrum of TAE (CD_3OD, 600 MHz).

Figure S5. HSQC NMR spectrum of TAE (CD_3OD, 600 MHz).

Figure S6. ^{1}H NMR spectrum of phyllemblin (CD_3OD, 600 MHz).

Figure S7. ^{1}H NMR spectrum of gallic acid (CD_3OD, 600 MHz).

Figure S8. ^{1}H NMR spectrum of cinnamic acid (CD_3OD, 600 MHz).

Figure S9. ^{1}H NMR spectrum of 1,3,6-tri-*O*-galloyl-β-D-glucose (CD_3OD, 600 MHz).

Figure S10. ^{1}H NMR spectrum of 1,2,3,6-tetra-*O*-galloyl-β-glucose (CD_3OD, 600 MHz).

Figure S11. ^{1}H NMR spectrum of 1,2,3,4,6-penta-*O*-galloyl-β-D-glucose (CD_3OD, 600 MHz).

Figure S12. ^{1}H NMR spectrum of p-coumaric acid (CD_3OD, 600 MHz).

Figure S13. ^{1}H NMR spectrum of chebulinic acid (CD_3OD, 600 MHz).

Figure S1. ^{1}H NMR spectrum of TAE by maceration (CD_3OD, 600 MHz).

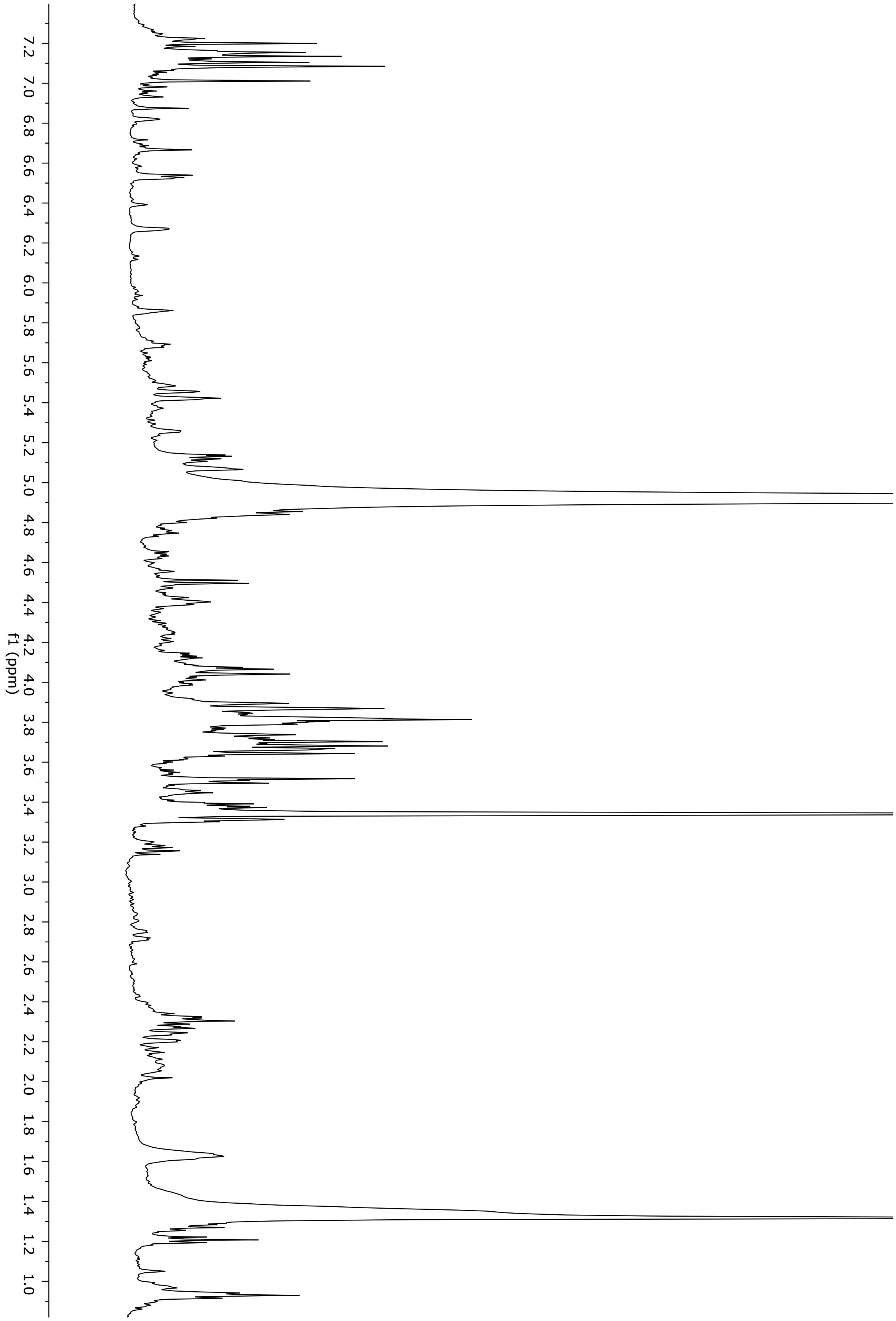

Figure S2. ^{1}H NMR spectrum of TAE by UE (CD_3OD, 600 MHz).

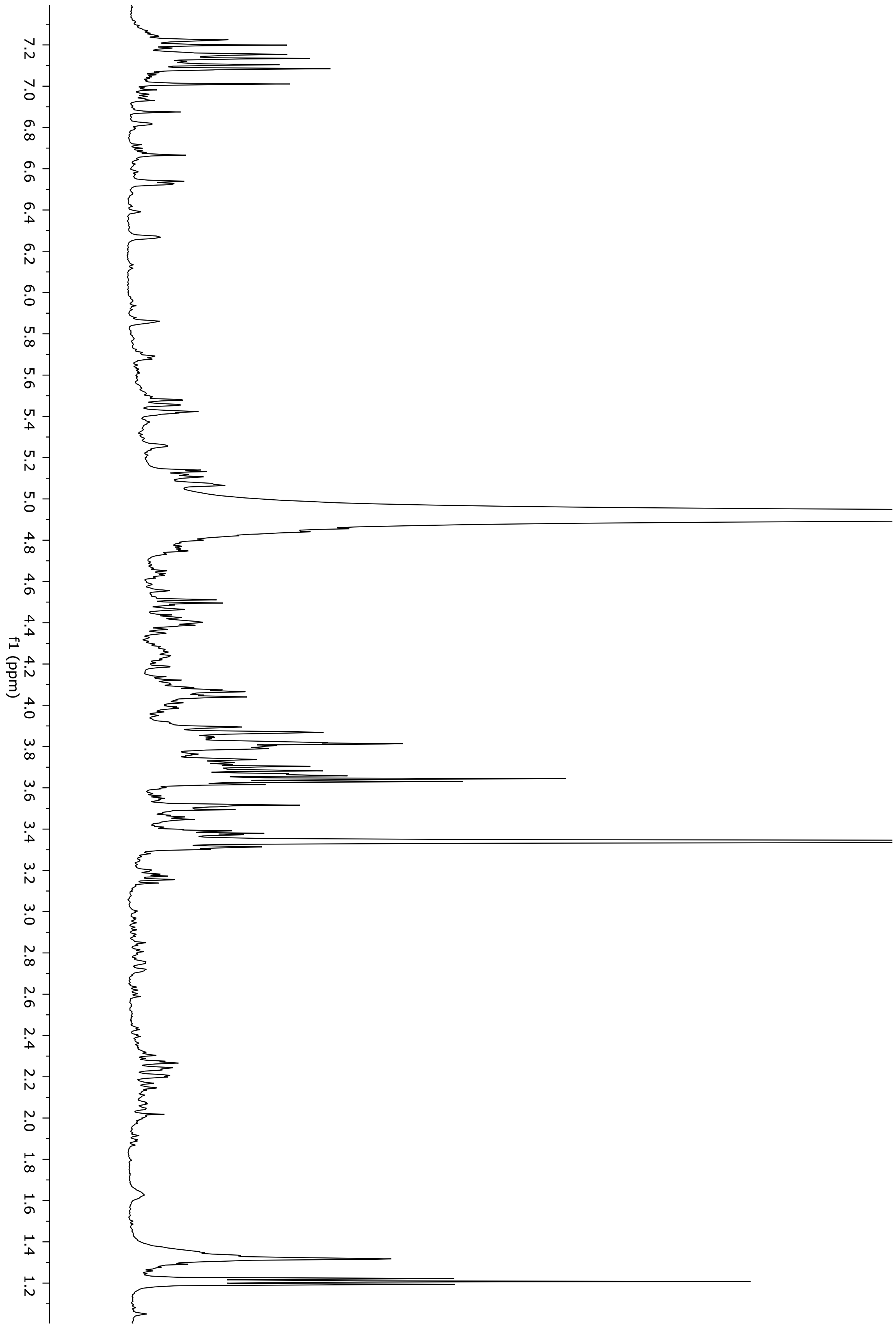

Figure S3. ^{1}H NMR spectrum of TAE by MAE (CD_3OD, 600 MHz).

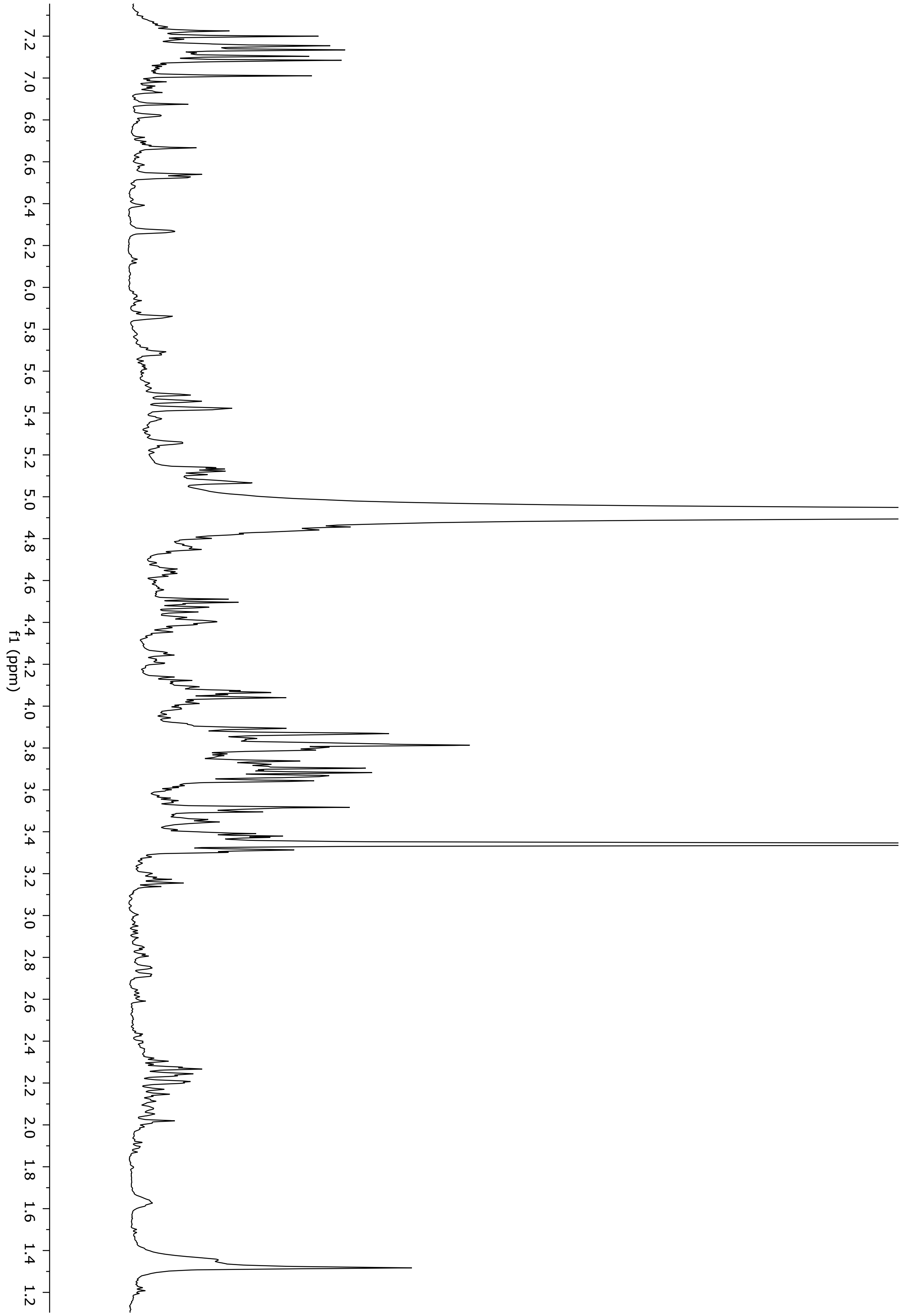

Figure S4. HMBC NMR spectrum of TAE (CD_3OD, 600 MHz).

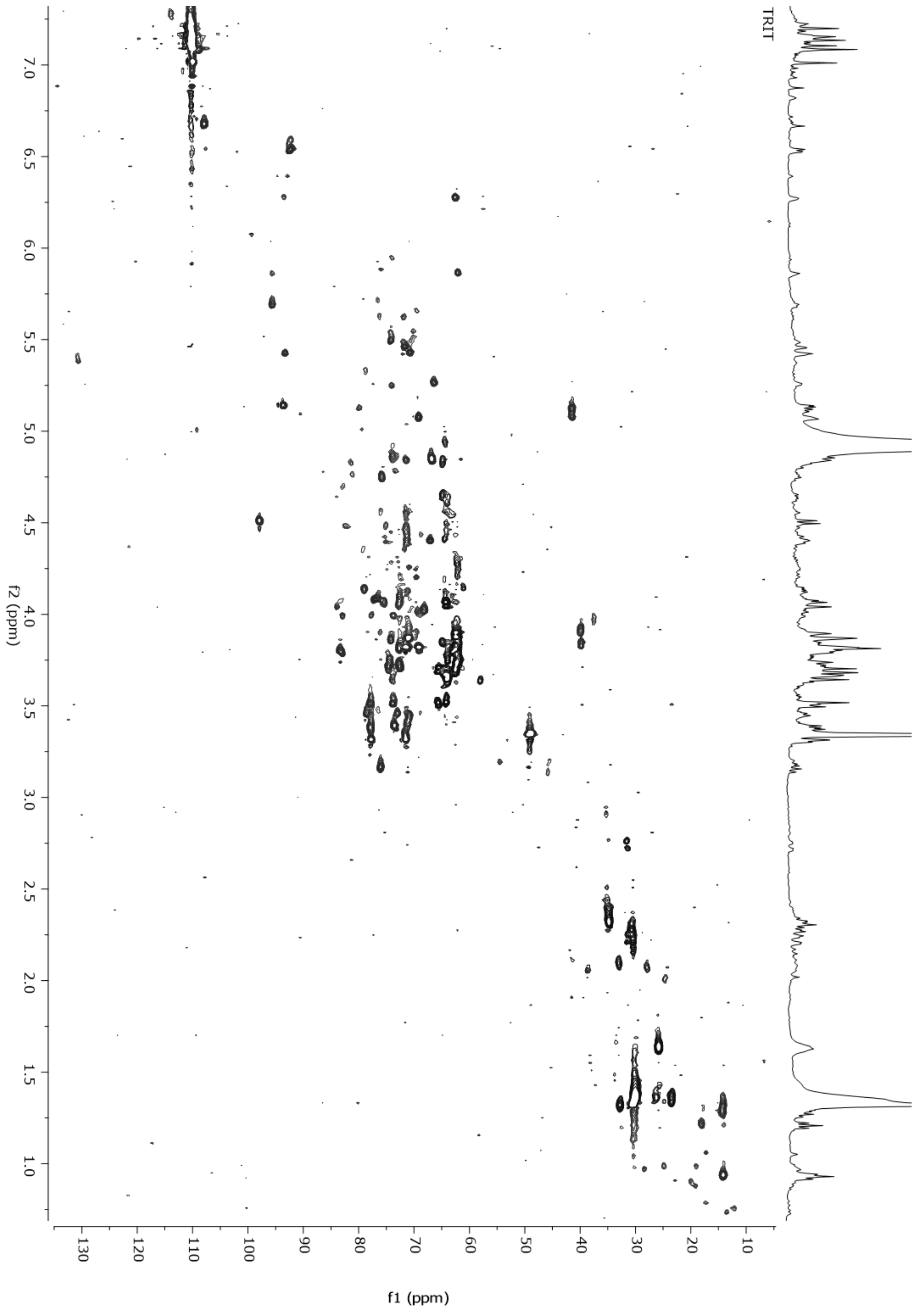

Figure S5. HSQC NMR spectrum of TAE (CD_3OD, 600 MHz).

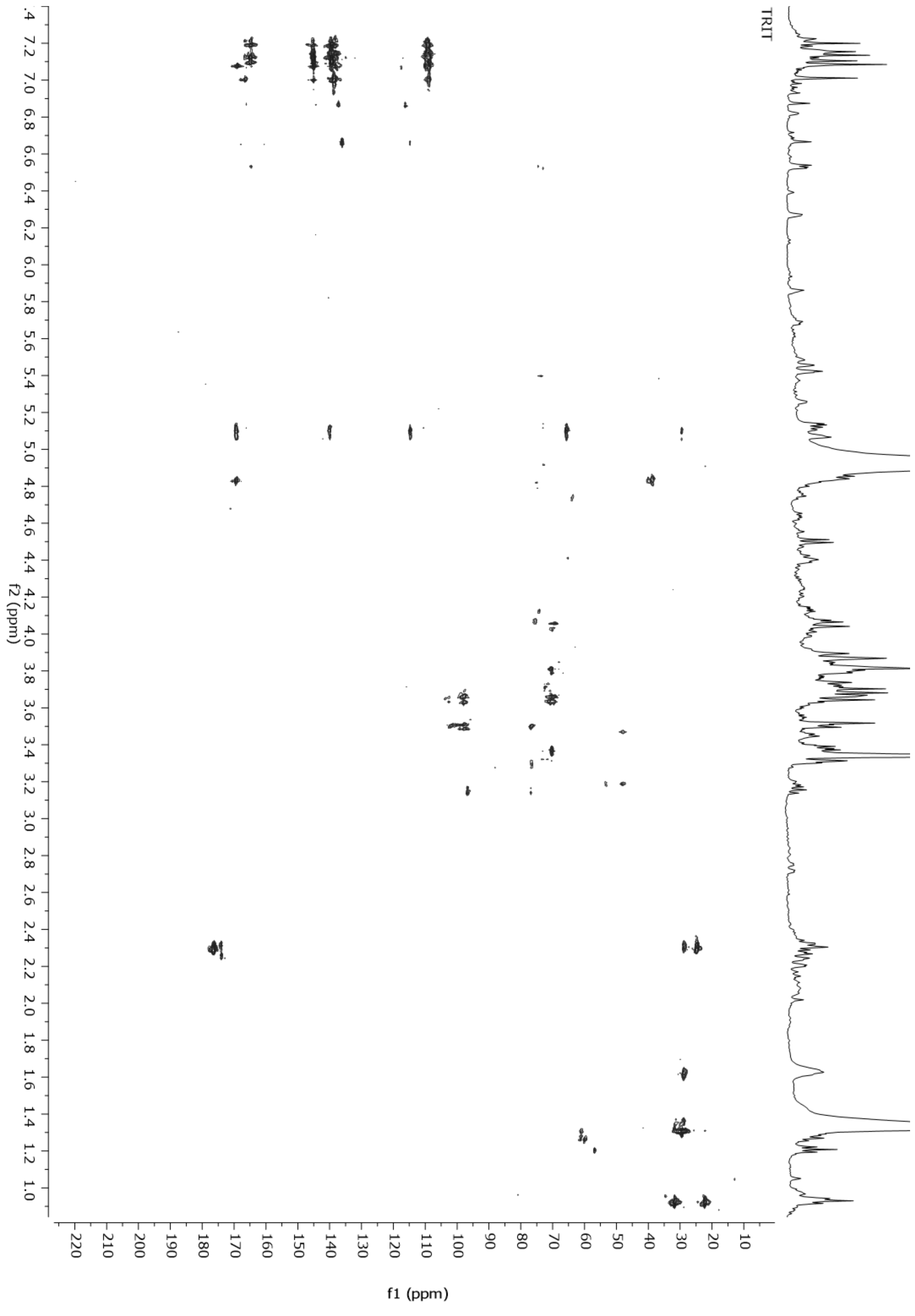

Figure S6. ^{1}H NMR spectrum of phyllemblin (CD_3OD, 600 MHz).

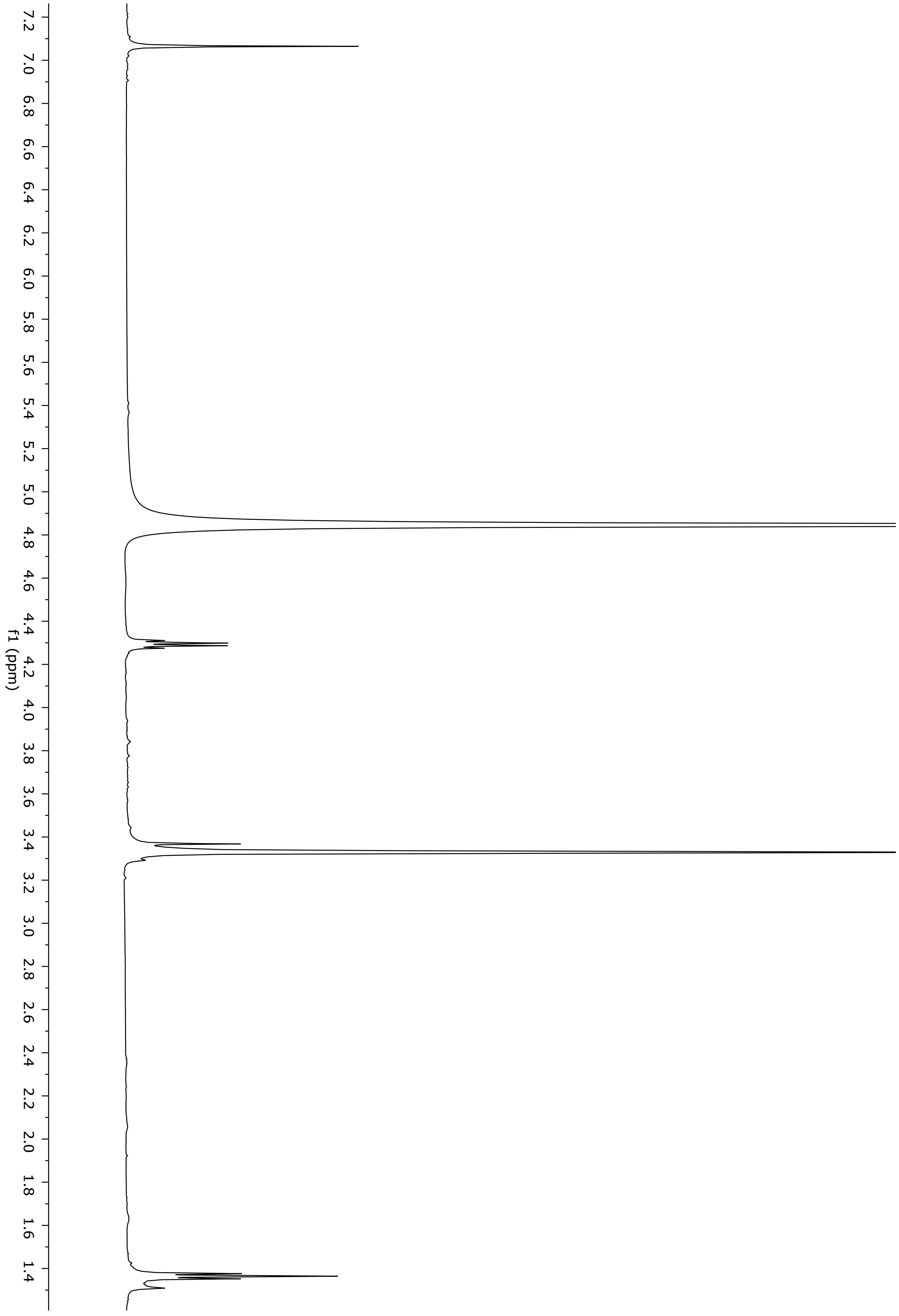

Figure S7. ^{1}H NMR spectrum of gallic acid (CD_3OD, 600 MHz).

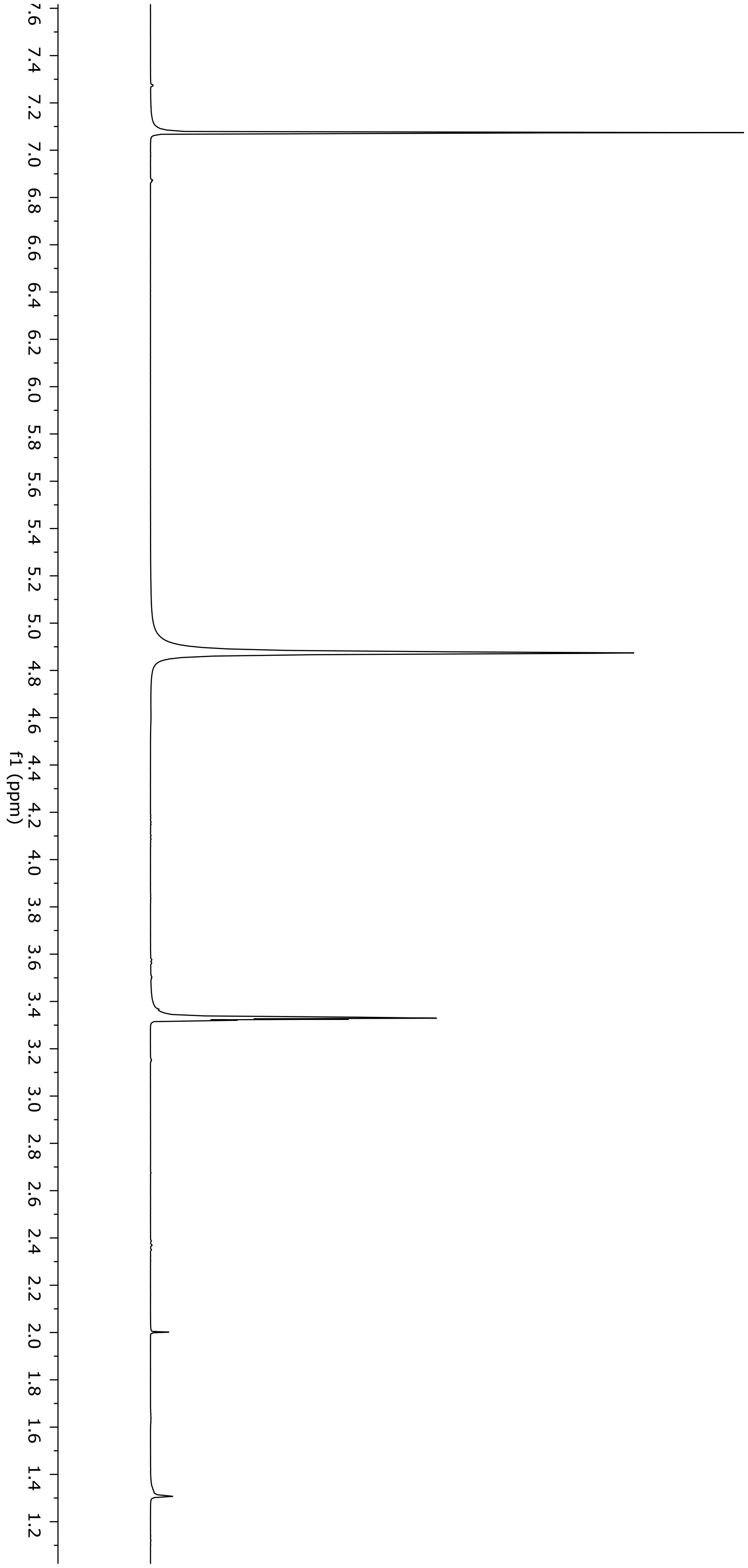

Figure S8. ^{1}H NMR spectrum of cinnamic acid (CD_3OD, 600 MHz).

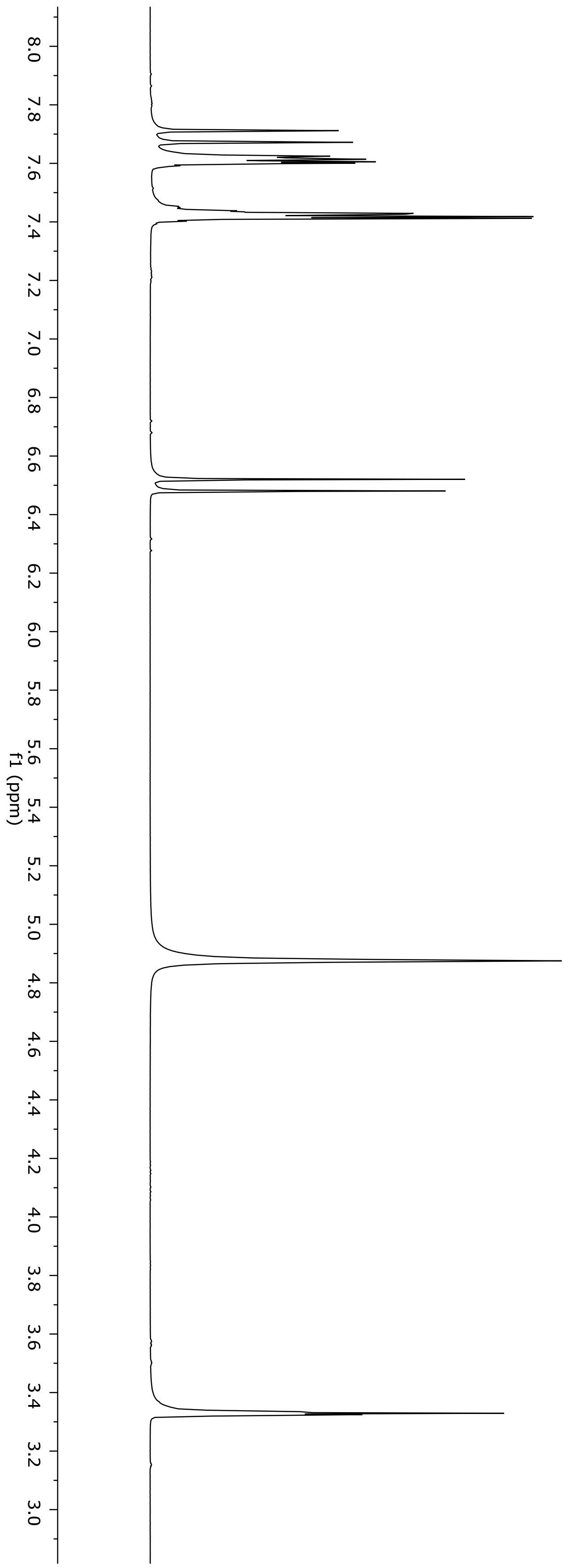

Figure S9. ^{1}H NMR spectrum of 1,3,6-tri-*O*-galloyl-β-D-glucose (CD_3OD, 600 MHz).

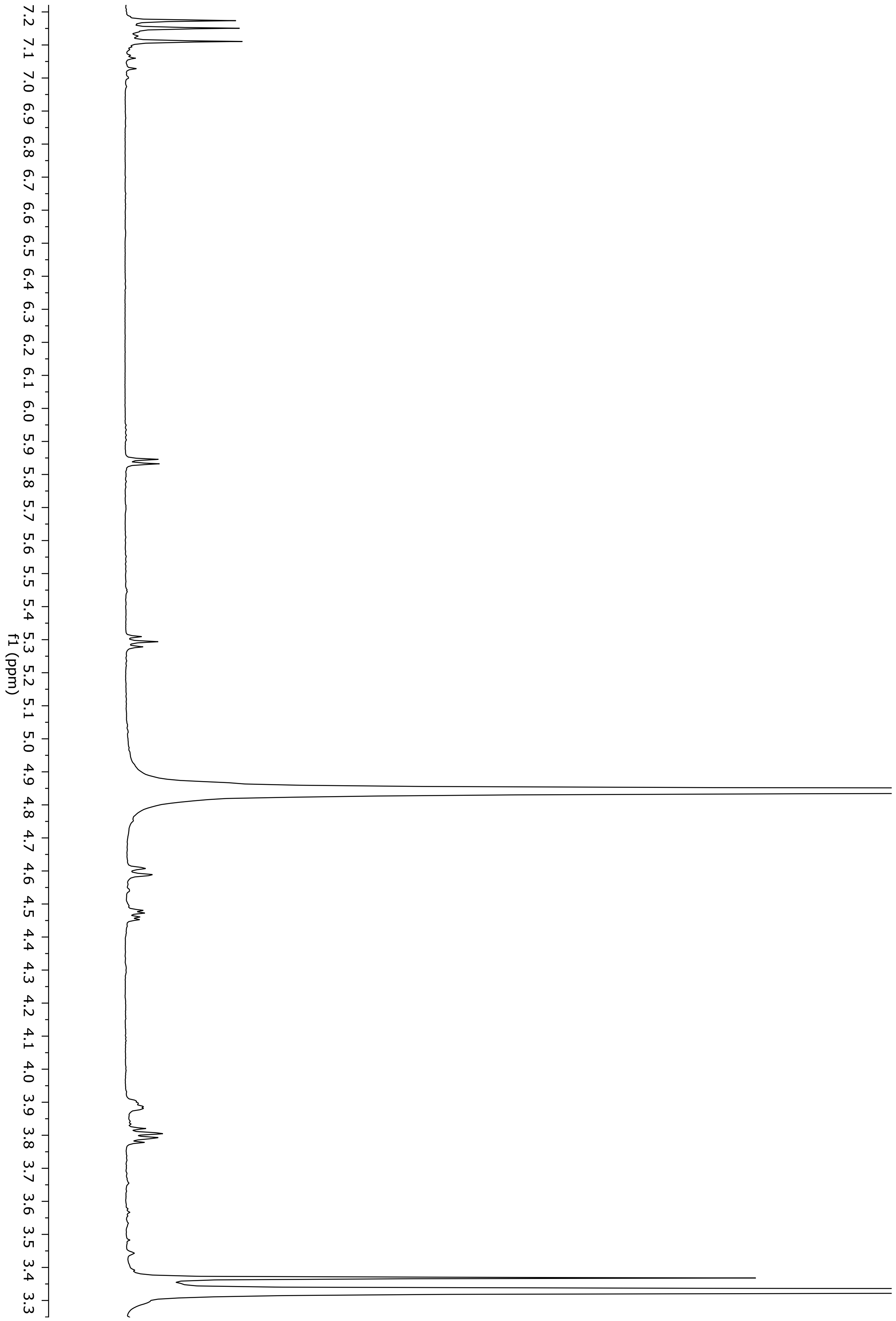

Figure S10. ^{1}H NMR spectrum of 1,2,3,6-tetra-*O*-galloyl-β-glucose (CD_3OD, 600 MHz).

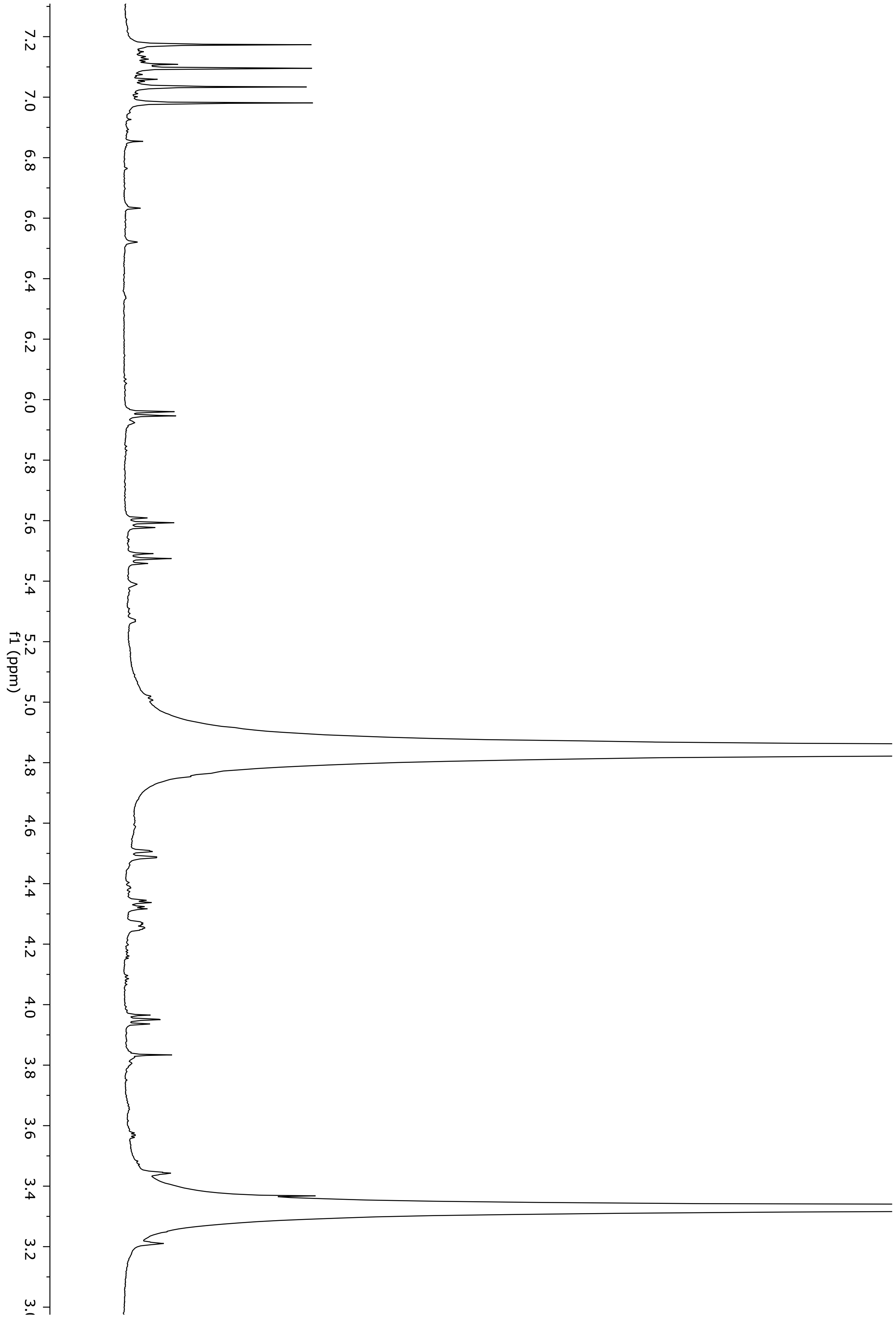

Figure S11. ^{1}H NMR spectrum of 1,2,3,4,6-penta-*O*-galloyl-β-D-glucose (CD_3OD, 600 MHz).

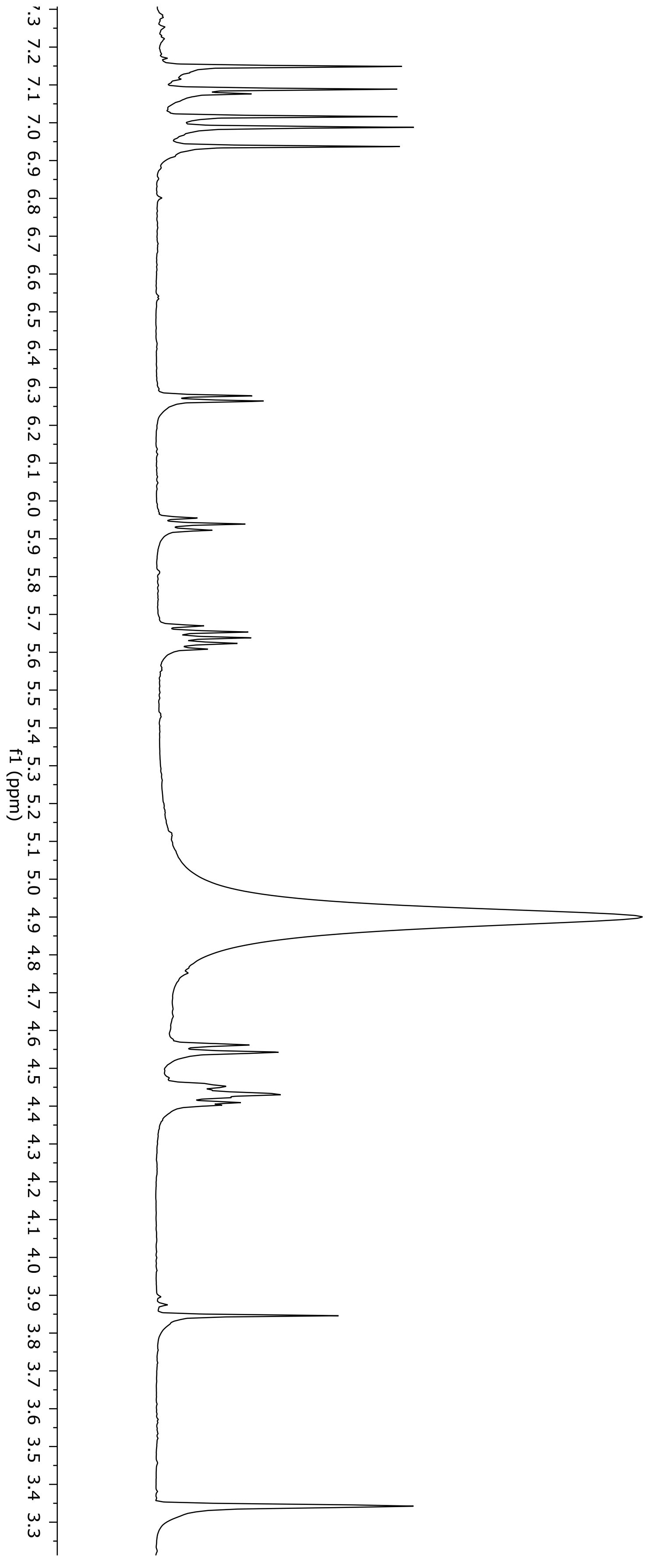

Figure S12. ^{1}H NMR spectrum of p-coumaric acid (CD_3OD, 600 MHz).

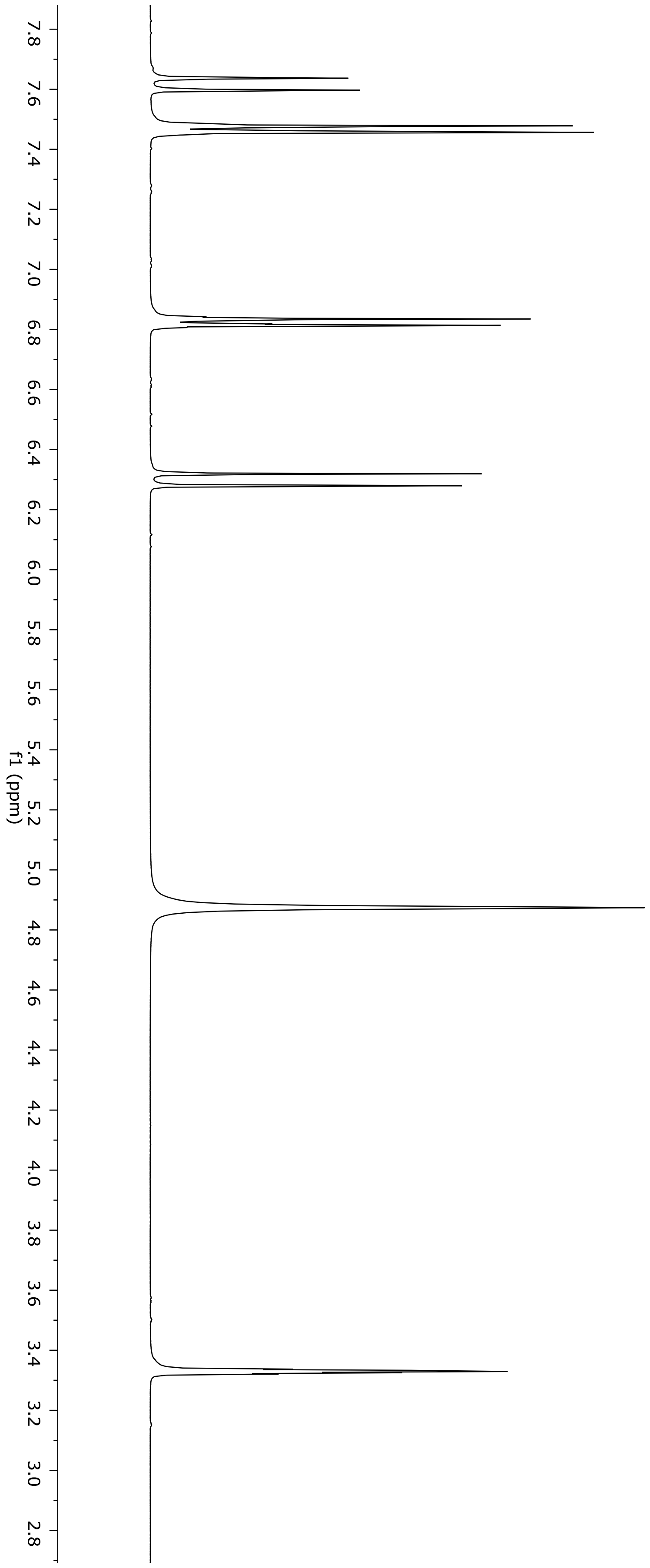

Figure S13. ^{1}H NMR spectrum of chebulinic acid (CD_3OD, 600 MHz).

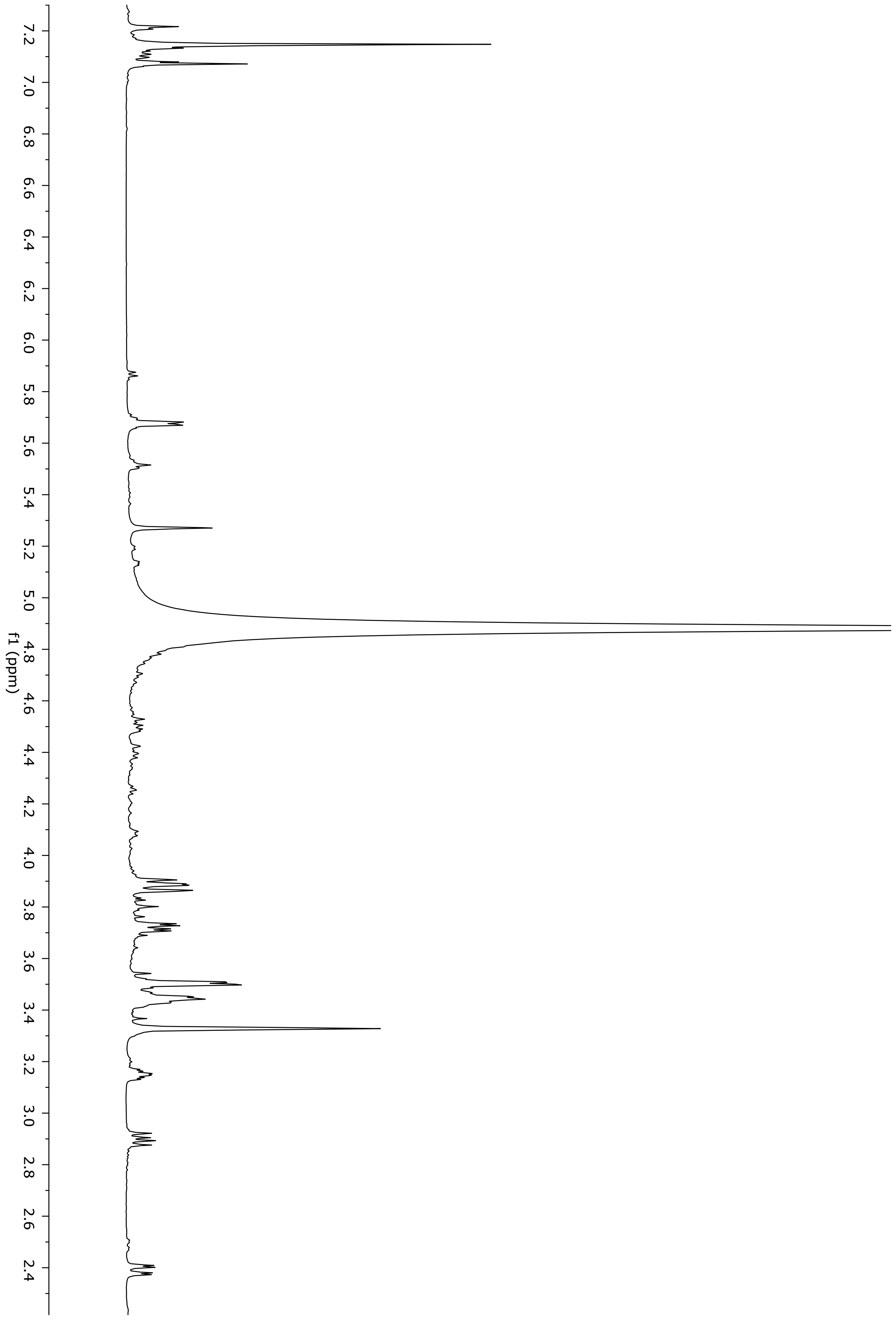

Supplement: Supplementary Materials — Figure S1: 1H NMR spectrum of TAE by maceration (CD3OD, 600 MHz). Figure S2: 1H NMR spectrum of TAE by UE (CD3OD, 600 MHz). Figure S3: 1H NMR spectrum of TAE by MAE (CD3OD, 600 MHz). Figure S4: HMBC NMR spectrum of TAE (CD3OD, 600 MHz). Figure S5: HSQC NMR spectrum of TAE (CD3OD, 600 MHz). Figure S6: 1H NMR spectrum of phyllemblin (CD3OD, 600 MHz). Figure S7: 1H NMR spectrum of gallic acid (CD3OD, 600 MHz). Figure S8: 1H NMR spectrum of cinnamic acid (CD3OD, 600 MHz). Figure S9: 1H NMR spectrum of 1,3,6-tri-O-galloyl-β-D-glucose (CD3OD, 600 MHz). Figure S10: 1H NMR spectrum of 1,2,3,6-tetra-O-galloyl-β-glucose (CD3OD, 600 MHz). Figure S11: 1H NMR spectrum of 1,2,3,4,6-penta-O-galloyl-β-D-glucose (CD3OD, 600 MHz). Figure S12: 1H NMR spectrum of p-coumaric acid (CD3OD, 600 MHz). Figure S13: 1H NMR spectrum of chebulinic acid (CD3OD, 600 MHz). [file 6976973.f1.pdf]
